# Male infertility as a late side effect of oncological treatment of Hodgkin lymphoma in childhood and adolescents and the possible prevention

**DOI:** 10.3389/fonc.2025.1655444

**Published:** 2025-10-27

**Authors:** Michelle Prusak, Katarzyna Brylka, Katarzyna Derwich

**Affiliations:** ^1^ Student Scientific Society, English Division, Faculty of Medicine, Poznan University of Medical Sciences, Poznan, Poland; ^2^ University of Medical Sciences, Institute of Pediatrics, Department of Pediatric Oncology, Hematology and Transplantology, Poznan, Poland

**Keywords:** Hodgkin lymphoma (HL), pediatric Hodgkin lymphoma (PHL), male fertility, male infertility, fertility preservation, oncofertility, pediatric, cancer

## Abstract

As the cohort of childhood and adolescent survivors of Hodgkin lymphoma (HL) continues to grow, the possibility of preserving long-term fertility has only amplified in the last decade. With the increasing success of pediatric cancer treatment outcomes, survivors now have the hope of having children of their own. This article critically reviews the relevant methods of gonadal function assessments and treatment therapies. It focuses on the most up-to-date methods of fertility preservation for male Hodgkin lymphoma survivors. Cure rates for HL remain among the best for pediatric cancers, with an overall success of 90-95% attributed to modern treatment. Although efforts to prevent gonadal toxicity continue to be of the utmost importance, ramifications of treatment have prevailed and sperm cryopreservation remains the gold standard method of fertility preservation. Pediatric physicians experience challenges in oncofertility that hinder the provision of high-quality fertility preservation care and patients endure a multitude of physical and psychological consequences that can exacerbate long-term fertility outcomes. Accordingly, fertility maintenance and protection must be considered extremely significant in all young male patients regardless of age, for maximal fertility quality and long-term potential for reproduction. Given the advancements in modern medicine, it is crucial to mitigate the long-term effects of treatments and prioritize fertility preservation options for young males to promote their reproductive autonomy.

## 1 Introduction

Reproductive problems are one of the main challenges that many patients have to address when dealing with the consequences of chemotherapy treatments for Hodgkin’s lymphoma. We summarize the diagnostic tools and assessments along with the potential treatment options that are available for pediatric patients. We highlight the importance of semen preservation and the current methods available in order to give male pediatric patients the opportunity for fatherhood in the future. Furthermore, we identify the next steps that may need to be taken in order to make this treatment process seamless and comfortable for the patients in hopes to provide them with the chance of even higher succession rates with higher fertility rates, and earlier detection.

One of the most common oncologic therapy complications is the impairment of fertility and reproductive health. This has been known to put a strain on romantic relationships, parenthood planning, and psychosocial well-being among pediatric cancer survivors. The issue of fertility preservation in pediatric patients presents a unique challenge especially since fertility preservation technologies have not been widely utilized for prepubertal patients with the long-term side effects and risks still being under investigation. Moreover, making informed decisions on parenthood goals and fertility preservation may be especially difficult due to the young patient’s age and subsequent development considerations, including the fact that the patient needs to be mature enough to understand the consequences of the decisions at hand. With this caveat, the discussion of potential cancer-related infertility is challenging yet incredibly necessary, as dictated by the American Society of Clinical Oncology (ASCO). Shared decision-making models that create discussions of sensitive topics at hand between patients, parents, and providers are currently being heavily investigated and are gradually becoming more and more prevalent in clinical practice.

## 2 Testicular anatomy and physiology

When examining the link between previous treatments for Hodgkin lymphoma and infertility, it is important to consider the underlying testicular anatomy and physiology. A major hurdle for cancer survivors is the impact of chemotherapy, radiotherapy, and, in some cases, testicular removal due to metastasis. All of these factors can result in both quantitative and qualitative deficiencies in spermatogenesis (1).

Spermatogenesis and steroidogenesis, the primary functions of the male reproductive system, are modulated by the hypothalamic-pituitary-gonadal (HPG) axis (2). Pulsatile release of hypothalamic gonadotropin-releasing hormone (GnRH) drives the number of hormones released from the anterior pituitary. High pulse frequencies stimulate the production of follicle-stimulating hormone (FSH), while low pulse frequencies promote luteinizing hormone (LH) secretion (3). LH stimulates the testicular release of testosterone, which is converted into its two active metabolites–estradiol (17β-estradiol) and 5ɑ-dihydrotestosterone (DHT)–via aromatase and is distributed to peripheral tissues (4). Weak-acting androgens including dehydroepiandrosterone and androstenedione bind to testosterone receptors and are converted to testosterone in peripheral tissues. Both testosterone and DHT bind to cellular receptors and regulate protein expression. FSH drives the production of androgen-binding protein (ABP), which allows more testosterone to cross the blood-testis barrier (BTB) and enter the seminiferous tubules where it stimulates their growth (5). FSH receptors are found on the Sertoli cells, and the binding of FSH induces and maintains normal sperm count and function (6). It is crucial for the normal physiologic process of spermatogenesis.

Sertoli cells also play a vital role by lining the seminiferous tubules and connecting to each other through tight junctions. This creates the essential blood-testis barrier, which, as previously discussed, separates the gametes within the seminiferous tubules from the immune system (7). As a consequence, seminiferous tubules are divided into adluminal and basal compartments with the basal zone being in constant contact with blood and lymph and the adluminal being completely disconnected from them. This separation is key to the immune privilege of the testis and has important implications for how different chemotherapy drugs interact with the testicular environment. Consequently, certain chemotherapeutic drugs may or may not be able to penetrate the BTB and therefore affect mature spermatids and spermatocytes. Notably, Sertoli cells that form the BTB are known to express very high levels of different drug transporters that depending on the chemotherapy regimens at hand may be associated with subsequent infertility (8).

In males, germinal stem cells are present in the testes from birth; however, sperm production, which is marked by nocturnal emissions and the presence of sperm in urine, does not begin until puberty. Pediatric Hodgkin lymphoma therapy has a deleterious effect on prepubertal testis, which are more vulnerable to cytotoxicity of various chemotherapy regimens (9). This vulnerability occurs because, in the prepubertal testes, early germ cells naturally degenerate before reaching the haploid stage, resulting in all germ cells being at the same developmental stage during chemotherapy. In comparison, mature testis contain germ cells that are in various developmental stages because germinal stem cells go through continuous self-renewal and differentiation into mature spermatozoa (10).

## 3 Hodgkin lymphoma

Hodgkin lymphoma is a malignant lymphoma of B-cell origin, which constitutes approximately 12-30% of all lymphomas. Although Hodgkin’s lymphoma is a relatively rare cancer, it is commonly diagnosed in patients between the ages of 15 to 19. Pediatric HL is typically defined as affecting patients ≤14 years of age and is typically more likely to be associated with male predominance within this age group. Previous infection with Epstein-Barr virus (EBV), immunodeficiency, and family history have been found to be predisposing risk factors (11). In contrast, adolescent/young adult Hodgkin lymphoma affects patients aged 15 years and above. This subtype has demonstrated no considerable gender proclivity and predominantly has a connection with a positive family history. In developing countries, HL is one of the most prevalent cancers that is commonly diagnosed in young adults with an average annual incidence of 2–3 per 100,000 individuals, which makes it a good precedent for investigating infertility associated with cancer treatment (12).

Despite the initial asymptomatic nature of Hodgkin lymphoma, the general symptoms may be subdivided into localized and systemic symptoms. Localized symptoms of HL arise due to compression of nearby structures by enlarged lymph nodes eliciting symptoms such as chest pain, dry cough, and shortness of breath, which are usually associated with the involvement of mediastinal lymph nodes. Splenomegaly and hepatomegaly are occasionally present. Systemic “B” symptoms include night sweats, unexplained weight loss >10%, and persistent fever. The definitive diagnosis in pediatric patients is usually made by surgical removal of the entire lymph node and sent for histopathological examination, which reveal Reed-Sternberg cells in the majority of HL entities. Utilization of PET and CT scans are useful in disease staging and detecting nearby structural involvement (12). In rare cases, Hodgkin lymphoma can present with testicular involvement. Malignant lymphoma of the testis generates 5% of all testicular malignancy and 1% of all lymphomas (13). Patients can possibly relapse and the malignancy can spread hematogenously or lymphatically. According to the results of the Childhood Cancer Survivor Study, the diagnosis of Hodgkin’s disease is associated with the worst testicular function (14).

## 4 Assessments of gonadal function

### 4.1 Semen analysis

The sperm analysis of Hodgkin lymphoma patients is evaluated on the basis of the World Health Organization (WHO) guidelines. In 2021, the WHO released their newest (sixth) edition of the WHO Laboratory Manual for the Examination of Human Semen. In this, the WHO highlights the criteria for assessing male reproductive health and diagnosing male infertility. Such parameters include sperm count, morphology, and motility. The suitable ranges were slightly adjusted compared to the previous fifth edition. Additional considerations new to the sixth edition includes the evaluation of semen odor, which was not included in previous renditions. Should a patient’s evaluation of sperm fall below the ranges, the patient is deemed to have low fertility potential.

Oftentimes, at the time of Hodgkin lymphoma diagnosis, patients have altered semen characteristics, higher sperm aneuploidy rates, low semen concentration and low sperm motility (15, 16). There is a clear correlation between impaired semen parameters and B symptoms at the time of diagnosis, which are often present in advanced stage HL as an expression of inflammation (16). Sperm quantity and quality are considered the best indicators of male fertility, and consistent poor motility is a good predictor of failure in fertilization (17, 18).

One large retrospective study investigated the semen quality in HL patient’s before and after different chemotherapeutic regimens. In so, it was determined that the majority of patients with Hodgkin’s lymphoma (~75%) prior to treatment showed normal semen readings. This was true in both early and late stages of cancer. However, in the later stages, there were some signs of lower semen volume and sperm count. Nevertheless, these patients still fell within the normal semen parameters set out by the most up-to-date WHO guidelines at the time. The researchers took into account the significance of the age group, especially if the patients had not yet reached sexual maturity. Most results indicated a decrease in sperm concentration and total sperm number during the early periods of the therapy cycles. There were also drops in progressive motility and increases in abnormal sperm forms. However, after more time passed post-treatment, the sperm quality returned to pre-therapy values. Another consideration was that these results are highly dependent on the number of treatment cycles that the patient undergoes. The less treatment cycles that the patient underwent, the higher chances of returning sperm quality back to pre-therapy levels (19).

It is important to consider that proper semen analysis can only be performed after puberty. While investigations on prepubertal spermatogonial testicular tissue is growing, assessments by testicular biopsies cancer patients are currently experimental and estimation of reproductive function in prepubertal boys depends on serum markers (20).

### 4.2 Hormonal analysis

#### 4.2.1 FSH and LH levels

Because follicle-stimulating hormone (FSH) and luteinizing hormone (LH) are considered the two major regulators of the steroidogenesis and spermatogenesis, they are important hormonal markers to evaluate. A rise in LH and FSH levels is one of the first indicators of damage to the germinal epithelium and marker of testicular dysfunction, due to inherent feedback regulation of both hormones. Chemotherapeutics have shown to strongly affect the levels of LH and FSH. Conventionally, FSH levels >10 IU/L are indicative of damage to spermatogenesis (21). One study assessed pre-treatment FSH and LH levels and reported significantly higher mean FSH and LH concentrations in patients diagnosed with HL when compared to healthy controls (22). FSH levels were found to be especially higher in male patients who received multiple rounds of procarbazine-containing chemotherapy regimens, boys who were late-pubertal, or young adults as compared to younger boys. Similarly, boys who had HL at more advanced stages or received more intensive treatment were found to have higher levels of FSH (20). While LH has been found to be more resistant to the damage of chemo- and radiotherapy than the germinal epithelium, multiple studies have cited elevated levels of LH in HL survivors pre- and post-treatment (20, 22, 23). One particularly surprising study found 33% of HL survivors had low LH concentrations post treatment, while the remaining patients did not display any elevations in serum LH (24). Contrastly, another study closely monitored LH levels post-treatment and found patients whose LH levels were initially within normal reference ranges had elevated levels over time (25). It can therefore be concluded that there is no current predictive prognostic serum levels of LH and FSH post-treatment for HL.

#### 4.2.2 Inhibin B and AMH levels

Sertoli cell markers include inhibin B and anti-Müllerian hormone (AMH). AMH is responsible for Müllerian duct regression in the male fetus as a part of the sexual differentiation process. AMH levels peak at 6 months, remain high during childhood, and slowly declines over a males lifetime. Because AMH is exclusively secreted from Sertoli cells, pediatric endocrinologists use it as a specific marker for testicular function during the male prepubertal period. It is a newly investigated serum marker in HL patients. Likewise, inhibin B has been identified as a marker for testicular function assessment since it regulates Sertoli cell function and spermatogenesis. The lower the level of inhibin B, the lower the sperm count levels will be in both healthy and subfertile men. Since inhibin B is a good gonadal marker for impairments, lower levels were indicated in long-term survivors of childhood cancer (26). One study found significantly lower mean inhibin B concentrations in HL patients when compared to healthy controls (22). Current data suggests that serum levels of inhibin B and AMH have been associated with cytotoxic spermatogenic failure that is related to radio- or chemotherapy (27). Two recent studies including patients treated with polychemotherapy have shown serum AMH levels below the norm for their respective ages (28). This can lead to low sperm retrieval rate, and subsequently, lower success of fertility.

#### 4.2.3 Testosterone levels

Testosterone is essential for maintaining the critical processes during spermatogenesis and overall male fertility. Testosterone regulates the maintenance of the blood-testis-barrier, completion of meiosis by spermatocytes, adherence of elongated spermatids to Sertoli cells, and the release of mature spermatozoa. Testicular function is greatly associated with appropriate testosterone levels. Due to the seminiferous tubule and germinal epithelium damage, many patients after treatment for HL have testicular volumes of 12 mL or less, which is clearly defined as a reduced testicular size compared to physiological size being 15 mL. Shrinking testes is a bodily response when there is not enough testosterone being secreted (25). Commonly, Hodgkin’s disease patients have lower total testicular volume, total testosterone <3.5 ng/mL and altered sperm count post-treatment (14). In so, pediatric HL patients often experience lower testosterone levels as compared to their equal counterparts, impairing appropriate testosterone production. One long-term study exhibited evidence of some pituitary downregulation due to the uncompensated decreases in testosterone concentration over time. However, it is important to consider that longitudinal measurements of total testosterone alone are not adequate for screening for testosterone deficiency for long-term lymphoma patients (29). Of note, testosterone levels are less evidently affected as compared to other markers (20).

#### 4.2.4 Estrogen levels

While estrogens have a direct adverse effect on spermatogenesis, a balance between serum estrogens and androgens is essential for maintaining normal serum parameters in male reproduction. Estradiol (17β-estradiol), the predominant form of estrogen, is transformed from testosterone catalyzed by aromatase in Sertoli cells. In the testes, estradiol contributes an important regulatory role in spermatogenesis, meiosis, spermatogonial proliferation, Sertoli cell function, spermiation, and sperm transport (30) Studies have shown that the G-protein-coupled estrogen receptor (GPER) expression by testicular peritubular cells is positively correlated with sexual maturation and male fertility (31). Conversely, reductions in the expression of GPER have been found to unfavorably affect spermatogenesis, as there is an inverse relationship between serum GPER levels and male fertility (30). A novel study investigated the effect of pediatric chemotherapy and the altered steroidogenesis and androgen/estrogen signaling pathways in *in vitro* matured testicular tissues of prepubertal mice. Researchers found an accumulation of estradiol in *in vitro* cultured tissues and concluded the surplus of estrogens could be delirious for sperm production (32). Interestingly, recent suggestions have investigated the potential use of selective estrogen receptor modulators to increase the sperm concentration and reduce estrogen levels in infertile men (33).

## 5 Treatment and its effects on fertility

Treatment for pediatric HL follows a multi-disciplinary approach which includes multi-agent chemotherapy, involved-site radiation therapy, and combined-modality therapy. A single efficacious treatment for pediatric HL is challenging to determine solely based on risk stratification and response criteria. Such risks include the tumor stage, severity of disease, and presence of B symptoms (11). Consequently, new treatment plans were introduced to reduce these risks. In 2021, the National Comprehensive Cancer Network (NCCN) released the first pediatric-specific guidelines for managing pediatric HL. Subsequent updates in 2023 have been implemented to address the rapidly advancing field of immune-oncologic therapies and the development of tailored approaches to radiotherapy. The guidelines underline the critical need to present patients with opportunities for clinical trials. Current clinical trials focus on limiting exposure to cardiotoxic chemotherapy, reducing exposure to radiation, and integrating novel agents for younger patients through collaborative efforts with the National Cancer Institute (NCI) National Clinical Trials Network (NCTN). Ultimately these guidelines aim to proactively address patient-reported symptoms and concerns related to quality-of-life by incorporating supportive measures to mitigate the potential long-term effects of treatment (34).

Studies have shown that platinum-based regimens and alkylating agents are the most important risk factor for infertility in males (35). Treatments with alkylating-agent-based chemotherapies in males have shown to likewise increase the risks of sexual dysfunction, azoospermia, and associated psychological issues (36). These profound effects of alkylating-agent chemotherapy in conjunction with radiotherapy affect the germinal epithelium in males. Therefore, cytotoxic treatment and radiation can potentially damage and deplete germ cells and spermatogonia, temporarily halting spermatogenesis and subsequently decrease the sperm quality (20). Recovery of fertility depends on the survival of stem cells, and if the entire stock of cells is depleted, then males are deemed to be infertile (37). Of note, while testicular Leydig cells can be affected, they are relatively resistant to treatment toxicity as compared to testicular germ cells. Therefore, survivors who are azoospermic after gonadal toxic therapy may maintain adequate testosterone production (38).

The most common manifestation of infertility is azoospermia, which is defined as the absence of complete lack of spermatozoa in the ejaculate. It is important to note that some pubertal males will have defective spermatogenesis prior to beginning treatment. The prepubertal testicle is likely equally or slightly less sensitive to chemotherapy compared with the pubertal testicle. Pubertal status is not protective of chemotherapy-associated gonadotoxicity (20).

### 5.1 Multiagent chemotherapy

Historically, MOPP (mechlorethamine, vincristine [Oncovin], procarbazine, and prednisone) approved in the 1960s and ABVD (doxorubicin [Adriamycin], bleomycin, vinblastine, dacarbazine) developed in the 1970s made long-term survival possible for patients with advanced HL. However, exposure to these treatments led to dose-related risks of infertility and cardiopulmonary toxicity (39). Studies have shown the cumulative effect to chemotherapy-induced imbalances in LH and FSH levels, and thus, Sertoli and Leydig cell damage. In efforts to reduce the unfavorable short and long-term side effects of therapy, hybrid regimens alternating MOPP, ABVD or derivative treatments were developed to lower the total cumulative doses of alkylators, bleomycin, doxorubicin and procarbazine. More recent pediatric trials have opted to use procarbazine-free regimens, strictly due to the fact that procarbazine is known to be gonadotoxic. To combat the gonadotoxicity, adjustments to these multi-agent regimens include ABVE-PC (doxorubicin [Adriamycin], bleomycin, vincristine, etoposide, prednisone, cyclophosphamide) in North America, and OEPA-COPDAC (vincristine, etoposide, prednisone, and doxorubicin - cyclophosphamide, vincristine prednisone, and dacarbazine) in Europe. OEPA-COPDAC is a treatment regimen derived from the standard OPPA-COPP (vincristine, procarbazine, prednisone, and doxorubicin - cyclophosphamide, vincristine, procarbazine, and prednisone). In 2022, a large, randomized multinational trial EuroNet-PHL-C1 examined the effects of treatment with OEPA and COPP (cyclophosphamide, vincristine, prednisone, and procarbazine) in conjunction with radiotherapy. The study found that male patients receiving COPP treatment had significantly higher FSH serum levels, lower inhibin B concentrations, and azoospermia as compared to their counterparts receiving COPDAC with dacarbazine. Of those treated with COPP, 19 of 23 males were azoospermic compared to those treated with COPDAC, whereby 0 of 22 males were azoospermic (40). By replacing the procarbazine in the therapy with etoposide and dacarbazine, the risk for gonadal toxicity in males have been significantly reduced. Of note, chemotherapeutic regimens that include more than one alkylating agent, often procarbazine with cyclophosphamide, chlorambucil, or nitrogen mustard have a high risk of permanent azoospermia if the treatment exceeds three cycles (20). Today, ABVD and BEACOPP (bleomycin, etoposide, doxorubicin, cyclophosphamide, vincristine, procarbazine, and prednisone) are the most commonly used multi agent therapies globally (41).

### 5.2 Radiation therapy

Pelvic radiotherapy is recommended for patients who have confirmed localization of residual disease after the end of systemic chemotherapy. Ionizing radiation can be used in combination with chemotherapy for early-stage and advanced-stage HL, especially with metastasis to the lymph nodes (35). However, radiotherapy has direct negative effects on male fertility. Exposure to radiation can produce destructive ramifications on the male testis, as it is the most radiosensitive organ in males. The germinal epithelium, including the spermatogonia, are more sensitive to radiation exposure than other cells. Leydig cells, on the other hand, are more resistant to damage and require a greater dose to reproduce the same damage (42, 43).

The dose of radiation is measured in Gray (Gy) units. Reversibility and irreversibility of damage is directly correlated to the dose of Gy given and the treatment period. The dose of radiation between 0.1 to 1.2 Gy has been linked to cause a reduction in spermatogonia while irreversible damage begins to occur at 4 Gy or greater (42, 44). The Euronet-PHL-C1 study reported that radiotherapy could be omitted in patients who responded appropriately to treatment (40). This further adds to the reduction of treatment-related gonadal toxicity.

### 5.3 Monoclonal antibodies

Significant progress has been made in recent years with regard to monoclonal antibody-based therapies. Brentuximab vedotin (BV) is a relatively new therapy with proven effectiveness in CD30 proliferative diseases, including HL and other non-Hodgkin types. While there are ongoing clinical trials to determine the toxicity and the efficacy of combining different chemotherapeutic sequences with brentuximab vedotin, it has promising outcomes. In a recent study, it was found that patients treated with chemotherapy in addition with BV had a 9.6%-point improvement in 3-year event-free survival as compared to conventional chemotherapy alone (45). In terms of fertility, for males treated with this drug, non-clinical studies have shown testicular toxicity. It has been linked to testicular atrophy and degeneration that is partially reversible (35). Because brentuximab is relatively new to the market, patients should be wary regarding conception. While fertility-preservation is not widely known, the U.S. Food and Drug Administration (FDA) approved the use of BV in combination with doxorubicin, vincristine, etoposide, prednisone, and cyclophosphamide for children over the age of 2 who have newly diagnosed, high-risk classical-Hodgkin lymphoma. This is the first pediatric approval of brentuximab vedotin (11).

Alternative monoclonal antibody therapies include human anti PD-1-monoclonal antibodies, namely nivolumab and pembrolizumab. Patients treated with anti PD-1 monoclonal antibodies are pretreated heavily with autologous stem cell transplantation (ASCT) and chemotherapy and are therefore highly susceptible to impaired infertility (35). Additional therapies include rituximab, a chimeric mouse/human IgG1k monoclonal antibody targeting the B cell surface antigen CD20 and bortezomib, a proteasome inhibitor. Anti-CD19 CAR T-cell therapy has also shown groundbreaking innovation in trials in HL. All of these immunotherapies are currently undergoing clinical trials for the treatment of pediatric HL (11). Because the consequences of these immunotherapies are not fully known or understood, there is insufficient data specifically regarding gonadal toxicity in males.

## 6 Preservation methods

Choosing ideal parameters of fertility involves a multifaceted approach and varies patient-to-patient. The preferred assessment tool used for male fertility is semen analysis, which is based on certain criteria such as sperm count, vitality, and mobility of spermatozoids. Semen analysis tests should be performed at least 3 months after completion of chemotherapy, as the duration of spermatogenesis lasts ~74 days. It is of utmost importance to propose semen collection and preservation prior to beginning chemotherapy in male patients, as there is a known risk for subfertility in HL patients and normal sperm preservation and quality control is essential for optimal fertility (35). Choosing the ideal preservation method should be made to minimize gonadotoxicity while maintaining therapy. Physicians treating pediatric patients with cancer should address and thoroughly inform patients of the possible risks of infertility prior to induction of gonadotoxic treatment. Preservation methods should be in accordance with the patient’s wishes.

### 6.1 Sperm cryopreservation and alternative methodology

Sperm cryopreservation (sperm-banking) is the established first-line recommendation for male patients of reproductive age. A simple acquisition method includes masturbation, as it is deemed the least invasive process as compared to other procedures. Patients who are unable to produce sufficient samples after masturbation are proposed to an alternative non-invasive method whereby post-masturbation urine samples are collected (46). More invasive techniques, such as testicular sperm extraction (TESE), penile vibratory stimulation and electroejaculation are preferred methods in patients with severe oligozoospermia, necrozoospermia, or ejaculation disorders and may sometimes be suggested due to the poor quality of sperm produced by cancer patients after diagnosis. However, these methods are less preferred as TESE requires surgical intervention with local or general anesthesia (42, 47). TESE also exposes patients to possible testicular complications, including inflammation, fibrosis, hematoma, and permanent devascularization (28).

Advancements in assisted reproductive therapies have shown promising success rates of fertilization, especially for patients with oligospermia. Oligospermia is defined as a sperm count less than 15 million per mL. Studies comparing fresh sperm to cryopreserved sperm have shown similar success rates of fatherhood, irrespective of etiology, with a generalized success rate of around 50% (42, 48). Additional assisted reproductive therapy techniques include the traditional approach of *in-vitro* fertilization (IVF). However, this method heavily relies on a large number of motile sperm, which can be challenging for male cancer patients with oligospermia. Comparatively, intracytoplasmic sperm injection (ICSI) is a method by which patients, even with abnormally impaired sperm count, are able to potentially reproduce. ICSI solves the problem of low sperm count and poor motility, since this modality requires a few motile sperm for fertilization (48). For this reason, ICSI can be particularly advantageous for cancer patients.

Obtaining sperm samples prior to the initiation of cancer therapy can be conducive to higher success rates of fertilization. According to the American Society of Clinical Oncology (ASCO), it is recommended that sperm be collected prior to initiation of therapy, as it is believed that the sample quality and sperm DNA integrity may be significantly jeopardized after a single treatment. ASCO guidelines recommend that sperm cryopreservation be offered to all postpubertal patients with a new diagnosis of cancer. It is necessary to obtain 2–3 ejaculates per patient since the quality of samples is typically diminished in cancer patients. Importantly, given the fact that the patient’s age needs to be taken into consideration, sperm banking is only going to be of benefit if the spermarche has been reached, which is usually the case at approximately 13–14 years of age. Subsequently, if spermarche and ejaculation have already been achieved, the patient’s age does not seem to affect the sperm quality (42). Chiefly, sperm cryopreservation is typically offered to patients who are at least Tanner stage 3 in pubertal development (47).

Other preservation methods are less commonly preferred in pediatric oncological patients. Of note, testicular tissue cryopreservation (TTC) has the greatest potential in pre-pubertal boys and adolescents with high risk of permanent azoospermia. The concept of harvesting testicular tissue is currently very experimental but in future may hold a lot of promise for the boys that haven’t reached spermarche yet due to the relative quiescence of prepubertal testes. TTC involves collecting testicular tissue via a transscrotal excision biopsy and cryopreservation by means of slow freezing techniques and subsequent isolation of spermatozoa from the thawed tissue while using intracytoplasmic sperm injection (ICSI) to complete fertilization. However, the theoretical risks of transplanting tumor cells and a low probability of spermatogonial cell post-thaw survival makes this method inferior to sperm cryopreservation. For this reason, further research and additional prospective development of experimental techniques for the maturation of spermatogonial stem cells into sperm is awaited upon (49–51).

## 7 Discussion

The end goal of fertility preservation in male oncological patients with Hodgkin lymphoma is the maintenance of fertility. Today, pediatric HL treatment predominantly focuses on retaining high cure rates and fertility while minimizing the toxic and long-term side effects. The impact of spermatogenesis in patients treated with HL regimens depends on several factors, including the type of treatment, when the treatment was performed and the time needed to recover testicular function. Despite the age of diagnosis and treatment, the attempt to preserve virility in male oncological patients is universal. However, due to the variety of preservation methods and consequential ramifications, it can be difficult for patients to attain. Studies in patients with lymphoma (both non-Hodgkin lymphoma and particularly Hodgkin lymphoma) have provided us, as physicians, with an unprecedented insight into cancer therapies and their correlation with fertility due to presenting at an earlier age which in turn offered a favorable prognosis. There is certainly a variety of treatment regimens, however, there is a clear causation link between older regimens with high doses of alkylating agents such as procarbazine, vincristine, cyclophosphamide, etc., and severe reproductive toxicity. Replacement therapies including etoposide and dacarbazine have significantly decreased infertility incidence rates in the pediatric population. However, there are currently limited studies that highlight the long-term success of reproduction. Studies primarily focus on biochemical markers and semen analysis as a means of measuring success rates of fertility, as opposed to birth rates (52). Further investigations can be made into lymphoma-specific biomarkers that could potentially predict infertility in response to specific cancer treatments.

Another point of contemplation should be that in general, lower fertility rates in Hodgkin’s lymphoma survivors could be at least partially attributed to the disease itself. Of note, oligospermia and abnormalities of sperm structure and function are apparent in some patients even before the treatment and are associated with advanced lymphoma stage and elevated erythrocyte sedimentation rate (ESR) (53). In a study conducted by the European Organization for Research and Treatment of Cancer Lymphoma Group (EORTC) and Groupe d’Etude des Lymphomes de l’Adultes (GELA), males with HL with generalized symptoms such as fever, night sweats, and increased erythrocyte sedimentation rate (ESR) were more vulnerable to infertility (35). For such reasons, it is imperative to take the disease itself into consideration when analyzing and determining infertility in patients.

Advancements in modern medicine have promising outcomes for fertility preservation in male HL patients. It is important to undergo the proper hormonal analysis and testing prior to- and post-treatment to protect the integrity of the male sperm. Albeit, prepubertal patient management requires more research and advancement in cryobiology and technology. Specifically, patients who have not yet achieved spermarche are at a disadvantage due to their immature reproductive system. To be able to see the results of cryopreservation is a long-term solution and the long-term effects have not yet been established. Hence, it is essential to speak to patients and include all information on longevity in the long-term treatment for HL. Statistically, many men undergoing cancer treatment do not participate in sperm cryopreservation practices and unfortunately, this procedure may not always be brought up for discussion in patients who are considered to be at risk for oncotherapy-related infertility. Risks of infertility and opportunities for fertility preservation should be openly explained and discussed before the initiation of treatment.

Pediatric HL survivors must endure a diversity of physical and psychological hurdles. Patients are frequently unaware of the extent of fertility risks and preservation methods as a consequence of a lack of education from physicians. Conversely, physicians often face challenges communicating such information to young-aged patients due to the stigma regarding parenthood in pediatric patients. Furthermore, in later years, male patients may be less likely to find a partner due to their medical history and may be less apt to desire children due to the psychological effects of the disease and consequential treatment. The perceived risk of congenital malformations amongst patients and their respective partners can likewise lead to a lower likelihood of pursuing conception. Therefore, patients and parents should be provided with all information at the time of diagnosis about the risk of infertility and the possible interventions to maintain biological reproductive potential. The American Society of Clinical Oncology (ASCO) recommends discussing oncofertility with patients and parents as soon as a definitive diagnosis has been made. Routine analysis with clinical counseling can facilitate shared decision-making within the multidisciplinary team. It is important that all patients are educated on their fertility options with respect to their personalized treatment.

## 8 Conclusion

The new evolving lymphoma treatment options have offered higher success rates of prolonging life expectancy in pediatric patients. The ultimate goal in Hodgkin lymphoma treatment is to ensure that there is still potential for fatherhood if the male pediatric patient decides to pursue it in the future. There are clear and definite changes in endocrine levels after chemotherapeutic treatment including but not limited to infertility, sexual dysfunction, azoospermia, and associated psychological issues. Conversely, there have been success stories which have shown high recovery status of semen quality two years after treatment. Oncofertility models of care must be heavily utilized in pediatric cancer patient’s care. The above listed new advances in semen preservation have made a significant impact on keeping the possibility of fertility alive in patients who may have thought they had to choose between health or fertility. Oncofertility models of care must be heavily utilized in pediatric cancer patient’s care.
